# Pharmacological therapy in the management of temporomandibular disorders and orofacial pain: a systematic review and meta-analysis

**DOI:** 10.1186/s12903-023-03524-8

**Published:** 2024-01-13

**Authors:** Giuseppe Minervini, Rocco Franco, Salvatore Crimi, Marco Di Blasio, Cesare D’Amico, Vincenzo Ronsivalle, Gabriele Cervino, Alberto Bianchi, Marco Cicciù

**Affiliations:** 1 Saveetha Dental College and Hospitals, Saveetha Institute of Medical and Technical Sciences (SIMATS), Saveetha University, Chennai, Tamil Nadu India; 2 Multidisciplinary Department of Medical-Surgical and Dental Specialties, University of Campania “Luigi Vanvitelli”, Via Luigi De Crecchio 6, 80138 Naples, Italy; 3 Department of Biomedicine and Prevention, University of University of Rome “Tor Vergata”, 00100 Rome, Italy; 4 Department of Biomedical and Surgical and Biomedical Sciences, Catania University, 95123 Catania, CT Italy; 5 Department of Medicine and Surgery, University Center of Dentistry, University of Parma, 43126 Parma, Italy; 6 School of Dentistry Department of Biomedical and Dental Sciences and Morphofunctional Imaging, University of Messina, Via Consolare Valeria, 1, 98125 Messina, Italy

**Keywords:** Temporomandibular disease, Drugs, Pharmacological treatment

## Abstract

**Background:**

Temporomandibular disorders (TMD) are manifested by soreness in the jaw joint area and jaw muscles, clicks or creaks when opening or closing the mouth. All these symptoms can be disabling and occur during chewing and when the patient yawns or speaks. Several classes of drugs are used to treat symptoms. This review aims to assess which drug suits the different signs.

**Methods:**

Pubmed, Web of Science and Lilacs were systematically searched until 01/02/2023. Clinical trials were selected that dealt with drugs used in temporomandibular dysfunction

**Results:**

Out of 830 papers, eight studies were included. The Meta-Analysis with Continuous Outcomes with Pre-Calculated Effect Sizes resulted in the rejection that there is intergroup variability (p.0.74).

**Conclusions:**

Treatment of orofacial pain is still a significant challenge for dentistry. We can conclude that there is no drug of first choice in the treatment of temporomandibular pain. However, the clinician must distinguish the type of pain and the aetioloic cause of the pain so that the patient can be treated and managed pharmacologically.

## Introduction

The temporomandibular joint (TMJ) is classified as a bicondylar diarthrosis. The joint is composed of the mandibular condyle and the glenoid fossa [1–4]. The TMJ is vital in guiding jaw movement and managing daily activities such as swallowing, chewing, and speaking [5–7]. Temporomandibular disorders (TMD) are a range of both musculoskeletal and degenerative conditions [8–12]. The leading causes of TMD are an altered position of the intra-articular disc or abnormal muscle hyperactivity. The main symptoms include joint clicks, limitation of movement, and facial muscle tension, known as orofacial pain [13, 14]. It is estimated that 25% of the population shows signs of TMD, while only a tiny percentage shows the need for treatment. Furthermore, the prevalence of symptoms is much higher in women than men [15]. The average age of patients with symptoms varies between 20 and 50 years. More than 70% of TMD patients show joint disc malposition as the cause. This condition is called an internal disorder. The progression of the disease is poorly understood; however, most affected individuals show a degenerative condition known as osteoarthritis or osteoarthrosis, depending on whether there is an inflammatory state. A clinical study on TMD patients with symptoms at the opening and closing of the mouth, on palpation, showed osteoarthritis phenomena at the joint level. Several studies have demonstrated by the magnetic resonance that the articular discs of asymptomatic patients in their normal anatomical position during movements have morphological change processes at the condyle and eminence level due to adaptive procedures [16, 17].

While in symptomatic patients, there is a critical alteration also at the bone level. The observed changes are abrasion and deterioration of the articular cartilage and bone remodeling. Treatment options vary according to the joint's internal imbalance severity and osteodegenerative phenomena. There are non-invasive or minimally invasive treatments for patients in the early stages of internal disorders [18–21]. While in advanced or complex cases, patients require joint replacements or invasive therapies [22]. The first phase of the pathology passes through the remodeling of the articular disc and bone heads. This process is one of adaptation and distributes excessive stress loads better. This is an adaptive process and therefore remains physiological. When the adaptive and remodeling capacity of the disc is exceeded, osteoarthritis phenomena occur. Significant alterations include alteration of bone components such as flattening of the joint eminence, decreased glenoid fossa, and flattening of the articular disc [23, 24].

Degenerative arthritis can result from a lack of adaptation or excessive or prolonged stress. All the alterations of the TMJ start from an imbalance and an alteration of the articular disc. The various pathological transitions from internal disorders to osteoarthritis are not understood; however, there is a correlation between the two conditions. The progression and onset of TMD are poorly understood; however, Wilkes has divided the passage into 5 phases. In stage 1, there is a painless click at the beginning of the opening and the end of the closing. There is a displacement of the disc forward and an inability of the disc to return to its original position. The bone bases appear unaltered. In stage 2, click symptoms and orofacial pain are present. The magnetic resonance shows a slight disc deformation and displacement forward; however, the disc in the maximum opening returns to the physiological position [25, 26].

Stage 3 is associated with frequent orofacial pain; jaw blocking during movement is more common.

There is a thickening of the articular disc. At the beginning of phase 3, the disc is recaptured but not entirely and in response, the disc deforms under the thrust of the condyle forward. During stage 4, symptoms are more persistent and include chronic pain and limitation in movement. The disc appears very thickened and is not recaptured during the maximum opening. There are also osteoarthritis phenomena. Stage 5 is the most advanced. Patients have persistent chronic pain, crackles, and movement restrictions. Non-invasive treatment of TMDs is the therapy of choice [27]. There are several non-invasive therapies available for the treatment of TMD. One of these therapies is drug treatment. TMDs affect a substantial portion of the global population, with prevalence rates ranging from 5 to 12%. The diverse nature of TMDs, which may involve myofascial pain, arthralgia, disc displacement, and osteoarthritis, contributes to the complexity of their clinical presentation. Patients often experience symptoms such as pain during mastication, restricted jaw movement, joint noises, and referred pain to the head and neck region. Moreover, TMDs can lead to additional comorbidities, including headaches, sleep disturbances, and psychological distress, underscoring the need for effective therapeutic approaches. Pharmacological interventions in the management of TMDs target the underlying pathophysiological mechanisms, aiming to alleviate pain, improve joint function, and enhance overall patient well-being. Non-steroidal anti-inflammatory drugs (NSAIDs) remain a cornerstone of pharmacotherapy, offering analgesic and anti-inflammatory effects through inhibition of cyclooxygenase enzymes. Muscle relaxants, such as benzodiazepines and cyclobenzaprine, act by reducing muscle hyperactivity and relieving muscle-related symptoms.

Tricyclic antidepressants (TCAs) and selective serotonin-norepinephrine reuptake inhibitors (SNRIs) have demonstrated efficacy in managing TMD-related pain, likely through their modulation of central pain pathways and neurotransmitter balance. Botulinum toxin injections, a relatively novel approach, target muscle hyperactivity and have shown promise in reducing pain associated with TMDs. Pharmacological interventions represent a vital component of the multifaceted management approach for TMDs. These interventions aim to alleviate pain, restore joint function, and enhance patients' quality of life. As our understanding of the pathophysiology of TMDs continues to evolve, it is imperative to critically evaluate the efficacy, safety, and long-term outcomes of various pharmacological agents.

This review aims to consider the different drugs used in the treatment of TMD [28]. In addition, a meta-analysis was conducted regarding the different pharmacological treatments of temporomandibular pain. The purpose of this systematic literature review with meta-analysis is to evaluate the main pharmacological treatments of pain caused by TMD. The purpose of this systematic literature review with meta-analysis is to evaluate the main pharmacological treatments of pain caused by TMD. In fact, the purpose of the meta-analysis is to evaluate which is the best pharmacological treatment of pain caused by temporomandibular disorders.

## Materials and methods

### Eligibility criteria

The following population (including animal species), Exposure, Comparator, and Outcomes (PECO) were used to determine the eligibility of all documents:

- P) Participants are patients with TMD
- E) Exposure consisted of patients with TMD treated with different types of drugs, nonsteroidal anti-inflammatory drugs, corticosteroids, antidepressant drugs, centrally acting muscle relaxants, anticonvulsants, benzodiazepines and to whom pain was assessed by VAS scale.
- C) Comparisons are patients with TMD and treated with placebo.
- O) Outcome is to evaluate the effectiveness of different types of drugs on TMD pain. As a secondary outcome is to evaluate the effectiveness on TMD treated with the different drugs compared with placebo.

Only papers providing data at the end of the intervention were included. Exclusion criteria were: 1) history of Temporomandibular joint (TMJ) trauma; 2) patients suffering from any inflammatory disorders or rheumatic diseases (e.g., rheumatoid arthritis, psoriatic arthritis); 3) patients with fibromyalgia; 4) patients with headache/migraine; 5) patients with a congenital abnormality or neoplastic conditions in TMJ region; 6) cross-over study design; 7) studies written in a language different from English; 8) full-text unavailability (i.e., posters and conference abstracts); 9) studies involving animal: 10) review article; 11) case report. Inclusion criteria are: patients with TMD; RCTs; observational studies; clinical trial.

### Search strategy

The study made use of major scholarly databases (PUBMED, WEB of SCIENCE, LILACS). The time period taken into account for the electronic search was from January 3, 2000, to January 2, 2023. Following the method outlined in Table 1, papers were systematically searched for in the databases of PubMed, Web of Science, and Lilacs. In addition, a manual search of earlier systematic reviews on the same subject was also done. The connector "AND" was used to unite the words "drug" and "temporomandibular disorders". MESH was utilized to assist with the web search (Medical Subjects Headings).

**Table 1:** Search strategy

***PubMed*** *"Temporomandibular disorders" AND "drugs"*
***Web of Science*** *(ALL = (temporomandibular disorders) AND ALL = (drugs)*
***Lilacs*** *temporomandibular disorders [Palavras] and drugs [Palavras]*

This systematic review was conducted according to Preferred Reporting Items for Systematic Reviews (PRISMA) guidelines and the Cochrane Handbook for Systematic Reviews of Interventions. The systematic review protocol has been registered on the International Prospective Register of Systematic Reviews (PROSPERO) with the following number CRD 42022316112 [29].

### Data extraction

Two reviewers (M.G.; R.F.) independently extracted data from the included studies using a customized data extraction on a Microsoft Excel sheet. In case of disagreement, a consensus was reached through a third reviewer.

The following data were extracted: 1) First author; 2) Type of study; 3) Sample; 4) Type of drugs; 5) Data used for meta-analysis; 6) Results.

### Quality assessment

The risk of bias in papers was assessed by two reviewers using Version 2 of the Cochrane risk-of-bias tool for randomized trials (RoB 2). Any disagreement was discussed until a consensus was reached with a third reviewer [30].

### Statistical analysis

The following factors were analyzed: VAS scale pre and after therapy. The mean values and standard deviation (SD) for each variable in each cohort were extracted. A meta-analysis was carried out when it was feasible. A 95% confidence interval (CI) for the mean difference was determined. Plots depicting forests were made to show the findings. Because of the high heterogeneity of the studies, the random effects model was used. Heterogeneity and the I^2^ statistic describes the percentage total variation across studies that is due to heterogeneity rather than change. The suggested interpretation of I^2^ is as follows: 0%–40% may represent low heterogeneity, 30%–60% may represent moderate heterogeneity, 50%–90% may represent substantial heterogeneity and 75%–100% considerable heterogeneity.

The pooled analyses were performed using the software SPSS version 27 (IBM Corp. (2020). IBM SPSS Statistics for Windows (Version 27.0) [Computer software]. IBM Corp). In this meta-analysis, used Pre-Calculated Effect Sizes models was used in order to assess the variances between the VAS scale before and after therapy with different drug classes. he reported summary statistics were calculated as random-effects models based on the assumption of heterogeneity between studies. Pooling was done according to the DerSimonian and Laird method, using inverse variance.

The *p*- value was set at 0.05 The Risk ratio between the two groups was measured. The Ta et Dionne study was divided into Ta et Dionne in which it evaluated the effects of naproxen and Ta et Dionne 1 in which the effects of celecoxib were evaluated.

## Results

### Study characteristics

**Fig. 1:**
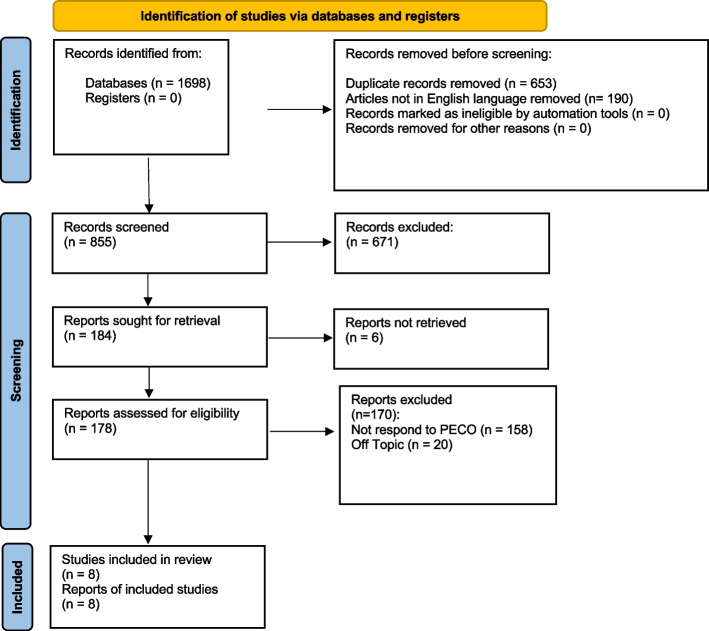
Prisma flowchart

At the end of the research, 1698 studies were identified. During the first screening phase, 190 articles were eliminated because they were not in English, precisely 130 from PubMed, 15 from Web of Science, and 45 from Lilacs. In addition, 668 items were excluded because they were duplicates. Ultimately, clinical trials and randomized trials were selected through special filtering. Therefore, 671 articles were excluded. In the final stage, 178 articles were selected, and abstracts were read. In the final stage of selection, 170 articles were excluded. One hundred fifty-eight did not meet the PECO, and 20 articles because they were off-topic (they treated oral-facial pain or did not meet the inclusion criteria) (Fig. 1). The selected studies come from various parts of the world and are heterogeneous regarding drug use. They consider a heterogeneous population of age. In the studies reviewed, adults were considered apart from Arabshahi's study, which evaluated children. In this systematic review, 531 patients were assessed; the population is heterogeneous regarding the type of TMD.

### Main findings

Ta and Dionne studied the pharmacological efficacy of NSAIDs. They performed a double-blind, randomized, placebo-controlled study in which they administered one group of celecoxib (100 mg twice daily), the second group of naproxen (500 mg twice daily) and the placebo control group for six weeks. This study showed that naproxen effectively reduces orofacial pain symptoms due to TMD compared to celecoxib. The study Singer [31] evaluated the efficacy of different drugs on gold facial myogenic pain thanks to a double-blind, randomized and controlled study. Thirty-nine subjects were recruited, including 35 women and four men, with orofacial pain due to TMD for at least three months and marked distress on palpation of the chewing muscles. Patients were treated for pain with one of the drugs to be tested: placebo, diazepam, ibuprofen, or a combination of diazepam and ibuprofen. Muscle pain, maximal opening and limitation of the movement were assessed at times 0 at two weeks and four weeks, respectively. Pain, as measured by a visual analogue scale, was significantly decreased in the diazepam and diazepam plus ibuprofen groups but not in the ibuprofen or placebo groups. Analysis of variance showed a significant pharmacological effect for diazepam but not ibuprofen, indicating that pain relief was attributable to diazepam. No significant changes were observed in muscle tenderness, interincisal opening or plasma beta-endorphin level [31, 32].

The study by List [32] evaluated the effectiveness of injecting an intra-articular dose of morphine. Fifty-three patients with joint pain were recruited. This randomized, double-blind study evaluated patients before treatment and a follow-up one week after treatment. The pain intensity was recorded using the VAS scale at the mouth's maximum opening and resting position. Intra articulaar injection was made into one TMJ containing either 1.0 mg morphine-HCl, 0.1 mg morphine-HCl, or saline (placebo). The pain was recorded in a diary three days before surgery and five days after. Pain assessed by VAS was significantly reduced 1–10 h after injection. The VAS score was lower in the 0.1 mg morphine group than in the 1.0 mg morphine group (*P* < 0.043) and in the placebo group (*P* < 0.021) [33]. Arabshahi's study evaluated the effects of corticosteroid injection into the temporomandibular joint in children with idiopathic arthritis and TMJ inflammation. Twenty-three children aged 4 to 16 years were recruited and received intra-articular corticosteroid injections (triamcinolone acetonide [*n* = 16] or triamcinolone hexacetonide [*n* = 7]). Pain and maximum incisal opening before and after treatment were evaluated as benchmarks. From the follow-up results of 13 patients with joint pain, 10 had complete resolution of symptoms with a significance of *P* < 0.05. All patients showed a reduction in mouth opening. After injection, the maximum aperture improved by 0.5 mm in 10 patients with a significance of *p* = 0.0017. Post-injection oedema occurred in only two patients [34]. In the Alstergren research, 22 patients (29 joints) with specific or nonspecific temporomandibular joint (TMJ) arthritis received a single intra-articular glucocorticoid (GC) injection. At follow-up appointments, 2–3 or 4–6 weeks following therapy, the impact on subjective symptoms, clinical signs in the craniomandibular system, and joint aspirate concentration of neuropeptide Y-like immunoreactivity (NPY-LI) were assessed. The medication improved the symptoms and clinical indicators in patients with the particular inflammatory joint condition, and 2–3 weeks later, the TMJ level of NPY-LI decreased. After 2–3 weeks, there was also a variable clinical improvement and NPY-LI level decrease in the patients with unspecific inflammatory joint illness, but not statistically significantly [35]. Several studies show the analgesic effect of tricyclic antidepressants in chronic pain. The study of Rizzatti-Barbosa showed that amitriptyline in the dose of 25 mg daily decreased symptoms in patients with chronic orofacial pain. While increasing the amount to 50–75 mg/day showed no substantial analgesic effects. The quantities of tricyclic antidepressants used to treat pain are much lower than those used to treat depression [36]. Another class of antidepressants used are SSRIs. They were introduced in the 1980s and have become the most widely used drugs for treating depression. This study of Kimos was to assess gabapentin's analgesic effects on myalgia. Fifty participants were randomly assigned to one of two research groups in this 12-week randomized controlled clinical trial: 25 received gabapentin, and 25 received a placebo. Palpation Index, VAS-measured pain, and a VAS-measured impact of myalgia on daily functioning were the outcome measures used (VAS-function). Thirty-six subjects completed the trial. Clinically and statistically, gabapentin was more effective than a placebo in reducing patient-reported pain, masticatory muscle hyperalgesia, and the impact of myalgia on daily functioning (Gabapentin = 51.04%; placebo = 24.30%; *P* = 0.037; gabapentin = 67.03%; placebo = 14.37%; *P* = 0.001; gabapentin = 57.70%; placebo = 16.92%; *P* = 0.022) [37].

The study of Gilron evaluates the administration of pregabalin in the wider variety of neuropathic pain etiologies in this multicenter experiment. Pregabalin was administered to 256 participants in this enriched enrolment randomized withdrawal trial in a single-blind, flexible-dose for four weeks when stable concomitant analgesics were permitted. A total of 157 patients were randomized and treated, double-blind, to receive either pregabalin (*n* = 80) or a placebo (*n* = 77) for five weeks, and 135 of them (65%) reported a pain improvement of at least 30%. Of the single-blind responders who were randomly assigned, 81% received a placebo, and 86% received pregabalin. At the double-blind endpoint, the pregabalin group's mean (SD) pain scores were 2.9 (1.9), and the placebo groups were 3.5 (1.7) (*P* = 0.002). These small but significant pregabalin-placebo differences were seen in each patient category with a diabetic peripheral neuropathy or postherpetic neuralgia diagnosis (*P* = 0.03) and those with other diagnoses (*P* = 0.02). Sleep disruption, Hospital Anxiety and Depression Scale Anxiety and Depression subscales, and other secondary measures showed significant variations. 28 out of 80 (35.0%) pregabalin users and 28 out of 77 (36.4%) placebo users discontinued the double-blind phase due to a noticeable increase in pain. In the single-blind stage, adverse events were consistent with the pregabalin tolerability profile. They resulted in the discontinuation of 9 patients, five in the placebo group and two in the pregabalin group [38] (Table 2).

**Table 2:** Main characteristics of the studies included in the present systematic review

First Author	Type of study	Sample	Type of drugs	Data used for meta-analysis	Results
Ta et Dionne,	Double blind, randomized	68 subjects with joint displacement	Naproxen,colecoxib, placebo	P:0.27; P:0.54	Two drugs are equally effective in reducing pain
Singer et al.,	Double blind, randomized	39 subjects	Placebo,diazepam, ibuprofen or a comination of the two drugs		Diazepam and the combination of the two drugs are more effective than ibuprofen
List et al.	Double blind, randomized	53 subjects	Intracapsular injection of morphine compared to placebo		the pain was significantly lessened
Arabshahi et al.	Randomized	23 children	Intracapsular injection of corticosteroid		Maximum opening and pain significantly decreased
Alstergren et al.	Randomized	22 patients	Intracapsular injection of corticosteroid		symptom improvement not statistically significant
Rizzatti-Barbosa et al.	Randomized	20 patients (female)	Administration of amitryptiline	P:0.72	There were no statistically significant effects
Kimos et al.	Double blind randomized	50 patients	Administration of gabapentin		gabapentin has a statistically significant pain relief effect
Gilron et al.	Randomized	256 patients	Administration of pregabalin	P:0.01	Pregabalin had an efficacy on the reduction of pain

### Quality assessment and risk of bias

**Fig. 2:**
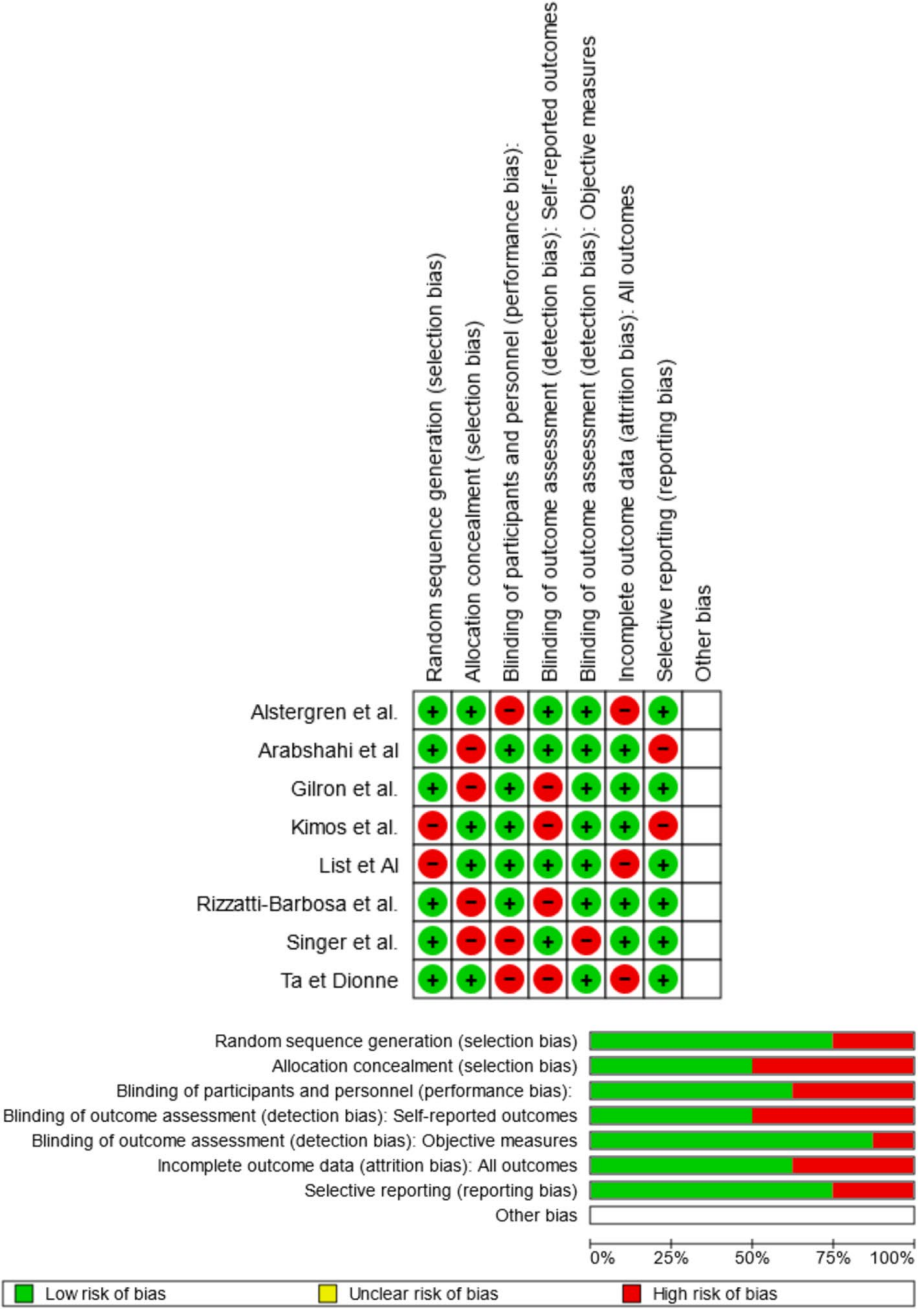
Risk of bias domains of the included studies

Using RoB 2, the risk of bias was estimated and reported in Fig. 2. Regarding the randomization process, 88% of the studies ensured a low risk of bias. However, 16% of the studies excluded a performance bias, but 84% reported all outcome data, 89% of the included studies adequately excluded bias in the selection of reported outcomes, and 50% excluded bias in self-reported outcomes. Overall, 6 of the eight studies were shown to have a low risk of incurring bias**.**

### Meta-analysis

**Fig. 3:**
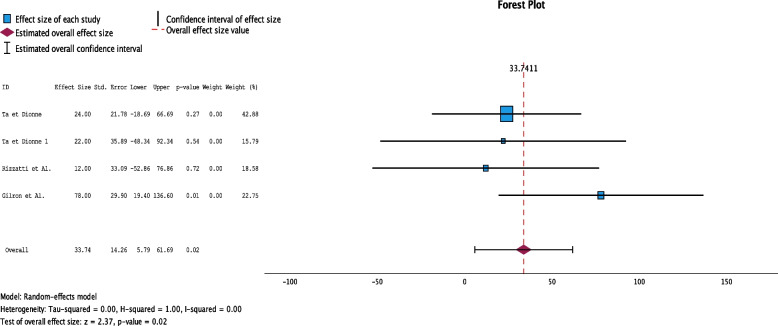
Forest plots of the analyzed studies showing correlation on different types of drug treatment for TMJ treatment. The study by Ta et Dionne evaluates the effect of naproxen before and after 6-week therapy; the study by Ta et Dionne 1 evaluates the effect of celecoxib; the study by Rizzatti et Al. evaluates the effect of gabapentin; the study by Gilron et Al. evaluates the effect of pregabalin. The *p* value was set at 0.5

The meta-analysis was conducted by SPSS software. Continuous Outcomes with Pre-Calculated Effect Sizes was performed because are continuous variables, using the p value at 0.05. In this meta-analysis, in order to statistically analyse the data, only studies with a control group and taking into account the VAS before and after therapy were taken into account, therefore the studies taken into account for statistics are 4. In order to conduct this meta-analysis, we evaluated and considered the change in VAS 6 weeks later in order to assess which is the best pharmacological treatment for TMD pain. The overall effect, reported in the forest plot (Fig. 3). The Forrest plot found no significant variation in pain symptomatology assessed by the VAS scale among the different therapies analyzed (Overall:33.47; C.I. 5.79–61.79). In Ta et Dionne we analyzed naproxen and celecoxib. In the study by Rizzatti Barbosa et al. we evaluated the effects of amytriptyline on the VAS scale. In the study by Gilron et Al. the effects of pregabalin were evaluated. The Meta-Analysis with Continuous Outcomes with Pre-Calculated Effect Sizes resulted in the rejection that there is intergroup variability (p.0.02). Effect Size Estimates for Individual Studies was reported in Fig. 3.

## Discussion

In light of the results of the meta-analysis and literature review, this article aims to evaluate which pharmacological treatment is most effective for the treatment of TMD pain. Therefore, we evaluated the main drugs used and compared their change in pain at 6-week follow-up. This meta-analysis showed no signigicative differences. Therefore, the clinician's choice on the type of drug treatment should be based on a number of variables including the patient's overall assessment, the presence of comorbidities. The following part will evaluate all the advantages and disadvantages of each drug, and then we can arrive at choosing a drug that can create fewer interactions and fewer side effects for the TMD patient.

NSAIDs inhibit cyclooxygenases and thus prevent the formation of prostaglandins. They were the most prescribed drugs for orofacial pain. NSAIDs treat patients with mild to acute TMJ inflammation, especially in disc dislocation cases, without reduction or acute trauma. These drugs must be taken for a minimum of 2 weeks. Several NSAIDs are used extensively in the dental field, such as ibuprofen, naproxen, diflunisal and ketorolac. The superiority of any NSAID over the others has not been demonstrated. The significant side effect is on the gastrointestinal level. NSAIDs can cause ulcers and bleeding in the gastric tract. Studies in the USA found that about 16,000 people die from gastrointestinal side effects [39–47].

Therefore, administering an NSAID to a patient with active gastrointestinal problems is not recommended. Naproxen and Ibruprofen appear to be the safest at the cardiovascular level. Ibuprofen also has fewer gastrointestinal effects. Another pharmacological possibility for patients at risk of gastric bleeding is using COX-2 inhibitors; however, patients should not have cardiovascular or cerebrovascular risk factors. NSAIDs can also interact with other types of drugs. For example, the clearance of Lithium decreased following the intake of NSAIDs. Therefore, there may be an increase in the serum concentration of Lithium and an increase in toxicity [48]. Combining NSAIDs with angiotensin conversion inhibitors or loop diuretics can cause acute kidney injury. NSAIDs reduce renal blood flow and the excretion of these drugs. Therefore, if NSAIDs are taken for more than five days, the effects of antihypertensive medications such as diuretics may be enhanced [49]. NSAIDs have an antiplatelet effect by inhibiting the synthesis of thromboxane. Therefore, they should be used cautiously in patients on anticoagulant therapy (e.g., warfarin) [50].

Widely used drugs are opioids, and their action against moderate pain is very effective. In the treatment of TMD, their use is mild to severe pain. The most widely used opioid drugs are codeine, oxycodone, and hydromorphone for severe pain. If the oral route is not preferred, one can opt for the fentanyl patch. The study did not show a variation between injectable and oral opioids [51].

Corticosteroid drugs have a chemical similarity to cortisol which is produced by the adrenal glands. These drugs are used to treat moderate to severe TMD. They block phospholipase A2, decreasing the production of prostaglandins and leukotrienes. Corticosteroids can be injected directly into the joint capsule or orally. Usually, intra-articular cortisone solutions are diluted with a local anaesthetic.

Other drugs used in the treatment of TMD are centrally-acting muscle relaxants. They are used and administered in patients with chronic pain. These drugs cause drowsiness and are therefore taken before going to bed. The most common muscle relaxants are carisoprodol, cyclobenzaprine, metaxalone and methocarbamol [52]. Antidepressive drugs are widely used for the management of TMD pain. Tricyclic antidepressant drugs and selective serotonin reuptake inhibitors appear to be the most important. Several studies evaluate the efficacy of tricyclic antidepressants in the management and control of chronic pain. Furthermore, patients with chronic pain also suffer from depression and sleep disturbances. The exact mechanism of action is not fully known; however, it is probably given by inhibiting the serotonin reuptake and noradrenaline at the synaptic level of the central nervous system. By blocking the reuptake at the back of the horn, there is an increase in the availability of these neurotransmitters, which block the transmission of pain. The drugs used are amitriptyline, nortriptyline and desipramine [53].

Adverse events of SSRI drugs are sweating, dizziness, blurred vision, and constipation. With an increase in the bioavailability of endogenous catecholamines, the administration of epinephrine can cause an overdose reaction. In addition, monoamine oxidase inhibitors, given together with tricyclic antidepressants, can lead to serotonin syndrome with fever, ataxia and severe hypertension. These drugs cause gastrointestinal problems, headaches, sexual dysfunction, dry mouth and sweating. These drugs should be used with caution. These patients need combined management with their treating physician. Anticonvulsant medications are often used for neuropathic pain. Their analgesic mechanism remains unclear. These drugs inhibit excessive neuronal activation. The sites of action are the voltage-gated ion channels. Gabapentin and pregabalin are used for orofacial pain. They have a chemical structure like GABA, the primary inhibitory neurotransmitter. However, none of these drugs acts on the GABA receptor [54].

Benzodiazepines are drugs used to treat sleep disorders and muscle disorders. These drugs are associated with tolerance and dependence; therefore, their long-term use is not recommended. These act on GABA receptors which mediate inhibitory transmission in the central nervous system. These drugs act on the chloride receptor, opening it and thus promoting neuronal hyperpolarization. They are mainly used as anxiolytics, but they also have their use as muscle relaxants. Their use for the treatment of epilepsy is also well established. Several clinical trials have evaluated the efficacy of these drugs in treating TMD compared to a placebo. These drugs have been discouraged due to adverse reactions such as sleepiness. Furthermore, these drugs show tolerance and dependence whereby the sudden discontinuation leads to symptoms including anxiety, agitation, restlessness, insomnia and convulsions. They should not be used in patients with myasthenia gravis and glaucoma. These drugs are degraded by the CYP 4503A4 and therefore show numerous interactions with other medications. Also, some agonist drugs of this cytochrome, such as grapefruit juice, can reduce the metabolism of benzodiazepines and thus increase their bioavailability. They are delicate drugs that experienced medical personnel must administer [55].

The main side effects of opioids are sedation, dizziness, nausea, vomiting, constipation, physical addiction, tolerance and respiratory depression. All these symptoms are accentuated in geriatric patients. The use of opioids with other central nervous system depressants, such as benzodiazepines, may have additive effects and cause increased sedation. Opioids are not recommended due to their tolerance and dependence. Therefore, the prescription of this category of drugs should be limited. Furthermore, no clinical evidence exists that long-term opioid therapy is more effective than other treatments [56, 57].

The structure of cyclobenzaprine is like tricyclic antidepressants. This drug is contraindicated in patients with hyperthyroidism, congestive heart failure, arrhythmias, and recent heart attacks. Low doses of cyclobenzaprine have positive effects on pain. Therefore, no more than 10 mg before bedtime, cyclobenzaprine is prescribed in low doses. The treatment plan should be 30 days, followed by a 2-week off period during which the patient's symptoms are evaluated. However, chronic therapies with cyclobenzaprine should be managed with the treating physician.

Therefore having evaluated all pharmacological mechanisms and side effects we were able to assess that as an effect on pain all drugs are effective. Moreover, given the side effects, the clinician should well categorize the pain and start through NSAIDs which are the drugs of first choice and with less side effects and then vary the therapy in case of failure.

### Limitations of this meta-analysis

The limitation of this meta-analysis is that we analyzed the different types of drugs together as we wanted to analyze which treatment was the most effective. However, we did not take into consideration the duration of treatment of each study. The limitation of this study lies in having evaluated different drug therapies for the treatment of TMDs.

## Conclusions

The study includes different types of pharmacological treatment for TMD and therefore we cannot state that there is a first choice drug for the treatment of pain. We can state that NSAIDs are the most widely used drugs. However, we can conclude from the review and the meta-analysis that NSAIDs are undoubtedly very effective drugs in the treatment of acute pain and are undoubtedly the safest drug class. Opiods are the substitute drugs for NSAIDs in the case of patients with previous gastrointestinal bleeding or in the case of acute moderate/severe TMJ pain Corticosteroids are always used for the treatment of acute moderate/severe pain, however, the first choice is an intra-articular injection. Myorelaxants are the drugs of choice either for acute contractions and/or contractures or are used to treat chronic pain. Another drug class used are antidepressants; they are used for chronic pain and in patients refractory to bite therapy. Anticonvulsants are drugs to treat neuropathic pain and thus chronic TMJ pain. Benzodiazepines are still drugs used in the treatment of chronic myofascial pain, however, they are drugs that need careful use and their usefulness also lies in alleviating sleep disturbances. In fact, the clinician should help himself with criteria such as DC/TMD for diagnosis and diagnostic classification.

Therefore, in conclusion, the clinician's skill lies in identifying the type of dysfunction and knowing how to choose drugs also on the basis of the patient's other comorbidities. Therefore, the gnathological framing of the type of dysfunction is fundamental and helps the clinician to choose the appropriate drug therapy. In addition, pharmacological treatment must be supported by functional therapy, physiotherapy and behavioural therapy.

## Data Availability

Data generated and analysed during study will be available from Rocco Franco upon reasonable request.
